# Quantitative magnetic resonance imaging measures as biomarkers of disease progression in boys with Duchenne muscular dystrophy: a phase 2 trial of domagrozumab

**DOI:** 10.1007/s00415-022-11084-0

**Published:** 2022-04-08

**Authors:** Sarah P. Sherlock, Jeffrey Palmer, Kathryn R. Wagner, Hoda Z. Abdel-Hamid, Enrico Bertini, Cuixia Tian, Jean K. Mah, Anna Kostera-Pruszczyk, Francesco Muntoni, Michela Guglieri, John F. Brandsema, Eugenio Mercuri, Russell J. Butterfield, Craig M. McDonald, Lawrence Charnas, Shannon Marraffino

**Affiliations:** 1 Pfizer Inc, Cambridge, MA USA; 2 Kennedy Krieger Institute, Johns Hopkins School of Medicine, Baltimore, MD USA; 3 Division of Child Neurology, Department of Pediatrics, University of Pittsburgh, Pittsburgh, PA USA; 4 Unit of Neuromuscular Disease, Bambino Gesù Children’s Hospital, IRCCS, Rome, Italy; 5 Cincinnati Children’s Hospital Medical Center, Cincinnati, OH USA; 6 University of Cincinnati School of Medicine, Cincinnati, OH USA; 7 Alberta Children’s Hospital, Cumming School of Medicine, University of Calgary, Calgary, AB Canada; 8 Department of Neurology, Medical University of Warsaw, ERN EURO-NMD, Warsaw, Poland; 9 Dubowitz Neuromuscular Centre, NIHR Great Ormond Street Hospital Biomedical Research Centre, Great Ormond Street Institute of Child Health, University College London, London, UK; 10 John Walton Muscular Dystrophy Research Centre, Translational and Clinical Research Institute, Newcastle University and Newcastle Hospitals NHS Foundation Trust, Newcastle, UK; 11 Children’s Hospital of Philadelphia, Philadelphia, PA USA; 12 Pediatric Neurology, Catholic University, Rome, Italy; 13 Centro Nemo, Fondazione Policlinico Gemelli IRCCS, Rome, Italy; 14 University of Utah School of Medicine, Salt Lake City, UT USA; 15 University of California Davis Health, Sacramento, CA USA

**Keywords:** Duchenne muscular dystrophy, Domagrozumab, MRI, Biomarkers, Imaging, Neuromuscular disease

## Abstract

Duchenne muscular dystrophy (DMD) is a progressive, neuromuscular disorder caused by mutations in the *DMD* gene that results in a lack of functional dystrophin protein. Herein, we report the use of quantitative magnetic resonance imaging (MRI) measures as biomarkers in the context of a multicenter phase 2, randomized, placebo-controlled clinical trial evaluating the myostatin inhibitor domagrozumab in ambulatory boys with DMD (*n* = 120 aged 6 to < 16 years). MRI scans of the thigh to measure muscle volume, muscle volume index (MVI), fat fraction, and T2 relaxation time were obtained at baseline and at weeks 17, 33, 49, and 97 as per protocol. These quantitative MRI measurements appeared to be sensitive and objective biomarkers for evaluating disease progression, with significant changes observed in muscle volume, MVI, and T2 mapping measures over time. To further explore the utility of quantitative MRI measures as biomarkers to inform longer term functional changes in this cohort, a regression analysis was performed and demonstrated that muscle volume, MVI, T2 mapping measures, and fat fraction assessment were significantly correlated with longer term changes in four-stair climb times and North Star Ambulatory Assessment functional scores. Finally, less favorable baseline measures of MVI, fat fraction of the muscle bundle, and fat fraction of lean muscle were significant risk factors for loss of ambulation over a 2-year monitoring period. These analyses suggest that MRI can be a valuable tool for use in clinical trials and may help inform future functional changes in DMD.

Trial registration: ClinicalTrials.gov identifier, NCT02310763; registered December 2014.

**Supplementary Information:**

The online version contains supplementary material available at 10.1007/s00415-022-11084-0.

## Introduction

Duchenne muscular dystrophy (DMD), the most common type of muscular dystrophy in childhood, has an estimated prevalence of 15.9 cases per 100,000 live male births in the United States [4, 5, 27]. DMD is an X-linked recessive disorder caused by mutations in the *DMD* gene and subsequent deficiency in the dystrophin protein. DMD is characterized by progressive muscle weakness and wasting, loss of ambulation, impaired airway clearance/ventilation, cardiomyopathy, and premature death [21, 22]. Current therapeutic options for the amelioration of signs and symptoms of DMD include physical therapy and treatment with corticosteroids. Other disease-modifying treatments that target specific dystrophin mutations and may be suitable in a small subset of eligible subjects include ataluren, which is only available in the European Union, eteplirsen, golodirsen, and casimersen, which are only available in the United States, and viltolarsen which is available in the United States and Japan [9, 11, 13, 18, 23, 30, 34]. Despite the development of new therapeutic options for DMD, the lack of robust biomarkers, the clinical heterogeneity of DMD, and, to a lesser extent, a lack of objective outcome measures that are sensitive to detecting disease progression and treatment effects, remain major challenges in clinical trials and drug development in DMD.

The North Star Ambulatory Assessment (NSAA) and functional tests with a time element such as the four-stair climb (4SC) and 6-min walk distance [15, 20, 28] are commonly used and validated functional assessments. However, functional changes as measured by these assessments develop slowly, which do not permit early detection of treatment-related changes. To address these challenges, several quantitative magnetic resonance imaging (MRI) measures have been proposed as biomarkers of disease progression for use in DMD clinical trials. These include MRI-derived measures of muscle volume, fat fraction, and T2 relaxation time; the latter is known to increase with fatty infiltration, inflammation, and edema [1, 2, 14, 16, 26]. Quantitative MRI measures offer the ability to characterize different aspects of the disease process over shorter time intervals, are well tolerated by most subjects without the need for sedation, and provide objective assessment of disease status that does not rely on subject performance.

We recently reported the results from a phase 2, randomized, double-blind trial to evaluate domagrozumab vs. placebo as a potential therapy for DMD [32, 33]. Domagrozumab is a humanized recombinant monoclonal immunoglobulin antibody subclass 1 (IgG1) that targets myostatin (GDF-8), a growth factor shown to negatively regulate skeletal muscle mass [3, 10]. In the *mdx* mouse model of DMD, inhibition of myostatin with RK35, a murine antibody equivalent of domagrozumab, led to increased muscle mass and strength, with decreased fat substitution and fibrosis [6, 17, 31]. The phase 2 study of domagrozumab did not meet its primary efficacy endpoint of mean change from baseline (CFB) in 4SC time at week 49 [32, 33]. However, there were favorable effects of domagrozumab vs. placebo on the mean percent change from baseline (%CFB) in thigh muscle volume and in muscle volume index (MVI) as detected by MRI, suggestive of target engagement [32, 33]. Here, we present an analysis of thigh MRI parameters collected during the phase 2 trial of domagrozumab and assess the potential for quantitative muscle MRI measures to serve as predictive biomarkers for concomitant functional changes in DMD.

## Methods

### Study design

This was an analysis of data from a phase 2, randomized, two-period (48 weeks each), double-blind, placebo-controlled, multiple ascending dose (5, 20, and 40 mg/kg) trial of intravenous domagrozumab in ambulatory boys with DMD (Clinicaltrials.gov: NCT02310763). A detailed description of the study design and inclusion/exclusion criteria has been reported previously [33]. In summary, participants aged 6 to < 16 years with clinically and genetically confirmed DMD, who performed the 4SC in ≥ 2.5 but ≤ 12 s at screening and were receiving a stable dose of corticosteroids for at least 6 months prior to screening, were eligible for the study. Participants were enrolled at 31 sites in 8 countries. Study endpoints included safety and tolerability of domagrozumab and mean CFB in 4SC time and NSAA total score at week 49 for domagrozumab vs. placebo. MRI measures were secondary (muscle volume measures) and exploratory (T2 mapping and fat fraction measures) study objectives.

### MRI imaging acquisition and analysis

#### Site setup and image acquisition

All imaging sites were trained on the study protocol and image acquisition procedures prior to the initial participant visits. Sites were provided with a scanning guide that covered all aspects of participant positioning, image acquisition, and quality controls. An MRI technologist from the central review facility (BioTelemetry Research, Rochester, NY) traveled to imaging centers to ensure consistency in imaging setup across all sites. Imaging facilities were required to submit phantom and healthy volunteer scans to demonstrate proper scanner setup and acquisition technique. Phantom scans using a Uniformity and Linearity (UAL) phantom, or an American College of Radiology (ACR) phantom, were performed to confirm that minimal spatial distortion occurred during the implementation of the MRI scanning protocol. The volunteer scans were inspected centrally to ensure compliance with the acquisition protocols and image quality requirements. After the volunteer test scans were approved the scanner and site MRI technologists were considered qualified to scan study participants. After scanners were qualified, quarterly scans of the UAL or ACR phantom were reviewed centrally to ensure no spatial distortion was introduced during the multi-year study.

Imaging protocols were developed to harmonize image acquisition processes across all imaging facilities using both 1.5 T and 3 T scanners. Scanning sequences and imaging protocols were designed and optimized to enable measurement of whole thigh muscle volume, whole thigh fat fraction imaging via Dixon imaging, and proximal thigh mean T2 relaxation time via T2 mapping. During image acquisition, unsedated participants were placed in a supine position inside the scanner, with the target leg supported off the table surface to avoid compression effects on muscle volume measurements. The same target leg of each participant (typically the right leg) was evaluated at each imaging visit (baseline and weeks 17, 33, 49, and 97). Additional details on the MRI setup and scanning protocol have been described previously [29].

For muscle volume measures, sites were instructed to acquire a proton density weighted axial fast spin echo, turbo spin echo, or spin-echo sequences. Images were acquired covering the thigh area from the acetabulum to the bottom of the patella using 5 mm slices with a 0 mm gap.

For Dixon imaging, sites were instructed to acquire axial 2- or 3-point Dixon scans using manufacturer provided sequences with the inherent body coil to allow acquisition of whole thigh images. Only sites that had manufacturer-supplied fat/water imaging sequences were required to submit Dixon scans for analysis. As in the proton density scan, images were acquired covering from the acetabulum to the bottom of the patella using 5–10 mm slices with a 0 mm gap.

For T2 mapping, scan acquisitions started from the top of the lesser trochanter and included ~ 7–10 cm of the proximal thigh. T2 mapping scans were acquired using a body/torso array coil and sites were instructed to acquire axial spin-echo scans with ~ 5 echoes. Copper sulfate belt phantoms were included in the field of view to allow quality control assessment of reconstructed T2 maps.

Imaging sites were instructed to use the same approved MRI scanner for all imaging time points for each patient. To account for minor changes in acquisition protocols between scanners, data were evaluated to look at change from baseline for each individual subject.

#### Image analysis

All images were evaluated at a central facility (BioTelemetry Research, Rochester, NY) as described previously [29]. Upon receipt, all images went through a quality inspection process to ensure images were compliant with the scanning guide, were high-quality acquisitions suitable for assessment (e.g., no significant artifacts), and that T2 phantoms were within the expected T2 relaxation ranges.

**Fig. 1:**
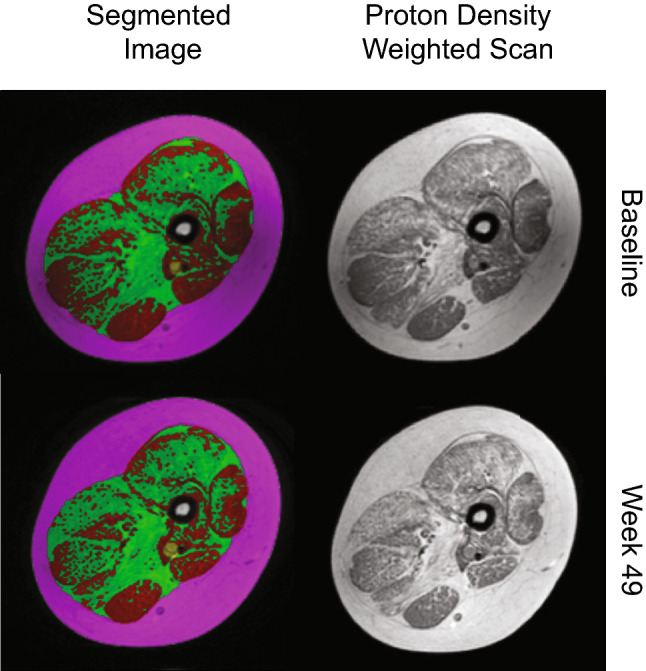
Representative example of a single slice of the thigh MRI at two time points. Representative image showing the proton density weighted scan at baseline and week 49. Both the acquired and segmented images are shown. Magenta region shows subcutaneous fat, red region show lean muscle, and green region shows inter/intramuscular fat. *MRI* magnetic resonance imaging

Segmentation algorithms based on fuzzy clustering and active contours [8, 25, 36] were used to initially segment images into bone, muscle bundle, and subcutaneous fat. The muscle bundle, or the cross-sectional region of the thigh excluding bone and subcutaneous fat, was further segmented into lean muscle and inter/intramuscular fat regions on the proton density weighted scans. Following automated segmentation, region of interest determinations were reviewed and adjusted by a study-trained, blinded imaging technologist. After the technologist review, segmented images were reviewed and adjusted by a study-trained, blinded, independent radiologist. The radiologist made the final determination on image readability and maintained responsibility for image segmentation quality and accuracy [8, 25, 36]. Examples of segmented images across a treatment period are shown in Fig. 1.

In addition to evaluating muscle volume, MVI was calculated. As previously described, MVI measures the fraction of the total thigh that is lean muscle [14] and is calculated as follows:

1 $${\text{MVI }} = \, \left( {{\text{muscle volume }}/ \, \left[ {{\text{muscle volume }} + {\text{ inter/intramuscular fat volume}}} \right]} \right) \, \times \, 100.$$

The T2 relaxation time was calculated on a pixel-by-pixel basis from the multi-echo T2 mapping scan using a non-linear curve fit, based on a mono-exponential decay model [1, 12, 29, 35]. The initial echo time was not included in the curve fit to minimize the effect of stimulated echoes on T2 calculation [12]. T2 maps were evaluated by both T2 relaxation time and percent of non-elevated voxels. The percent of non-elevated voxels was the percent of total voxels in the T2 mapping acquisition with a relaxation time < 55 ms [29]. Voxels with an elevated T2 relaxation time are more likely to represent fatty or inflamed tissue [1, 35]. The 55 ms threshold is elevated above what is observed in healthy muscle tissue [1, 35], which is consistent with its application here.

Water-fat images acquired by the Dixon imaging protocol were used to generate fat fraction maps. The fat fraction map was calculated as follows:

2 $${\text{`Fat image'}} / ({\text{`Water image'}} + {\text{`Fat image'}}).$$

Mean values from and T2 maps and fat fraction maps were evaluated in two regions of interest. The first region included the entire muscle bundle, whereas the second was limited to lean muscle.

### Statistical analysis

A mixed model for repeated measures analysis with terms for stratification factor, baseline MRI result, treatment, time, and treatment by time interaction as fixed effects, and participants as a random effect, was used to assess the difference in MRI measurements between domagrozumab and placebo groups at week 49. Change and percent change from baseline for each visit was attributed to the last dose received at the previous visit. Baseline was defined as the last pre-dose assessment collected at the screening visit. Unscheduled and early termination readings were excluded.

The relationships between MRI endpoints assessed at week 49 (%CFB in thigh muscle volume, thigh MVI, and inter/intramuscular fat volume; and CFB in mean T2 relaxation time of the muscle bundle, mean T2 relaxation time of the lean muscle, percent non-elevated voxels of the muscle bundle, mean fat fraction of the muscle bundle, and mean fat fraction of lean muscle) and functional endpoints (4SC and NSAA) assessed at week 97 were evaluated using simple linear regression. For these analyses, all participants from each treatment sequence were combined and only those participants with a week 97 functional assessment were included.

Additional analyses were performed to assess the relationship between MRI endpoints at week 49 and functional endpoints at week 97 using regression tree methods [7]. For each pairwise comparison (MRI vs. functional endpoint), a regression tree was constructed to “split” the MRI endpoint into two subgroups that yielded the smallest level of variability on the functional endpoint within each subgroup. Regression trees were also utilized to assess the relationship of both muscle volume and muscle quality together at week 49 vs. each of the functional endpoints at week 97.

Time to loss of ambulation was defined as the number of days on study until the first onset of an adverse event recorded as “Gait Inability.” Cox proportional hazards regression models were used to compare the time to loss of ambulation between participants above and below the median baseline value for each MRI parameter. Additional analyses assessed the time to loss of ambulation with a Cox model using each MRI parameter as a time-varying covariate. For all Cox models, age was included as an additional covariate. All *P* values are presented nominally without an adjustment for multiplicity, and therefore should be interpreted as exploratory analyses.

## Results

### Participants

Of the 120 participants who were included for analysis in the phase 2 study, all had at least one MRI assessment. In total, 118 boys had scans to measure change in muscle volume and muscle volume index, 114 boys had scans to evaluate T2 mapping changes, and 97 had Dixon scans to evaluate changes in fat fraction. The demographics and MRI characteristics of participants at baseline were generally balanced between the domagrozumab and placebo arms (Table 1).

**Table 1:** Participant demographics and baseline MRI characteristics^a^

	Domagrozumab (*n* = 80)	Placebo (*n* = 40)
Age, mean (SD), years	8.4 (1.7)	9.3 (2.3)
Weight, mean, (SD), kg	30.1 (8.6)	35.3 (14.4)
Height, mean (SD), cm	123.4 (8.4)	128.9 (10.0)
Muscle volume, mean (95% CI), mm^3^	1,047,597 (985,585–1,109,608)	1,079,792 (1,001,544–1,158,040)
MVI, mean (95% CI), %	69.9 (66.3–73.5)	64.6 (59.4–69.9)
Inter/intramuscular fat volume, mean (95% CI), mm^3^	513,728 (425,087–602,370)	725,946 (507,591–944,302)
T2 muscle bundle, mean (95% CI), ms	73.0 (70.2–75.8)	75.2 (71.5–79.0)
T2 lean muscle, mean (95% CI), ms	65.9 (63.8–68.1)	67.6 (64.6–70.5)
Percent non-elevated voxels, mean (95% CI), %	23.8 (19.7–27.8)	19.3 (14.2–24.3)
Fat fraction muscle bundle, mean (95% CI), %	39.1 (35.3–42.9)	45.2 (39.7–50.6)
Fat fraction lean muscle, mean (95% CI), %	22.1 (20.7–23.6)	23.4 (21.5–25.4)

For the domagrozumab group: *n* = 78 for muscle volume, MVI and inter/intramuscular fat volume; *n* = 75 for mean T2 measures and percent non-elevated voxels; *n* = 65 for mean fat fraction measures. For the placebo group: *n* = 40 for muscle volume, MVI and inter/intramuscular fat volume; *n* = 39 for T2 measures and percent non-elevated voxels; *n* = 32 for mean fat fraction measures. Age and race information were from screening visit, and weight and height information were from baseline visit

*CI* confidence interval, *MRI* magnetic resonance imaging, *MVI* muscle volume index, *SD* standard deviation

^a^Only subjects with baseline and at least one post-baseline value are included

### MRI analysis

#### Thigh muscle volume and muscle volume index

**Fig. 2:**
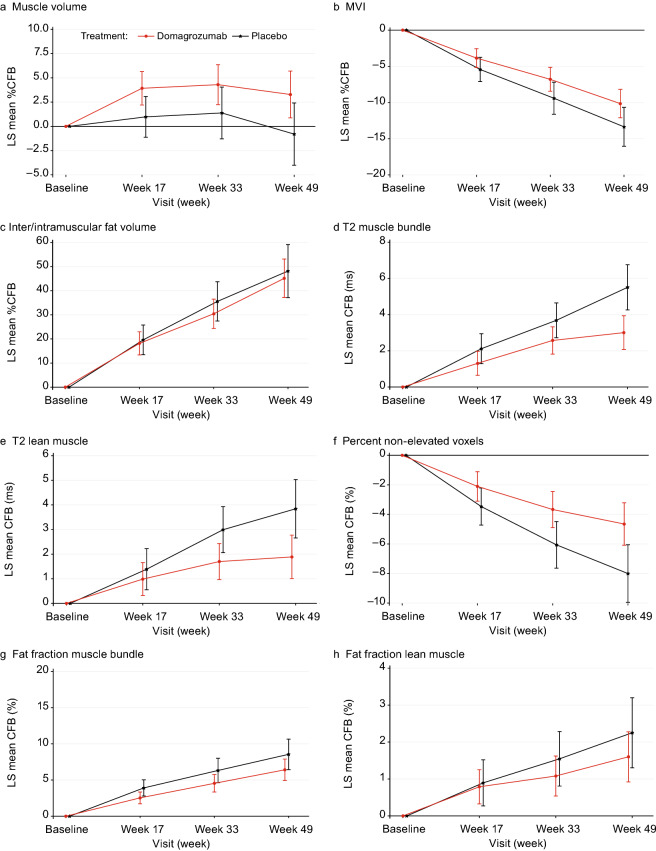
Results from MRI measures at week 17, week 33 and week 49. **a**–**c** Muscle volume, MVI and inter/intramuscular fat volume are derived from a proton density weighted MRI scan; **d**–**f** muscle bundle, lean muscle, and percent non-elevated voxels are derived from the T2 mapping MRI scan; **g** mean fat fraction of the muscle bundle and **h** mean fat fraction of lean muscle are derived from a Dixon fat fraction scan. *%CFB* percent change from baseline, *CFB* change from baseline, *MRI* magnetic resonance imaging, *MVI* muscle volume index

There was a significant increase in thigh muscle volume with domagrozumab compared with placebo at week 17 (difference 2.95%; 95% confidence interval [CI] 0.76, 5.13; *P* = 0.009) and week 49 (difference 4.09%; 95% CI 0.41, 7.77; *P* = 0.030; Table 2 and Fig. 2a). In addition, there was a significant increase in MVI with domagrozumab treatment compared with placebo at week 33 (difference 2.61%; 95% CI 0.15, 5.07; *P* = 0.038) and week 49 (difference 3.21%; 95% CI 0.13, 6.28; *P* = 0.041; Table 2 and Fig. 2b). There were no significant differences in inter/intramuscular fat volume between treatments (Table 2 and Fig. 2c).

**Table 2:** Mixed model repeated measures analysis of domagrozumab vs. placebo

Week	Participants (*n*)		Adjusted mean (95% CI)		Difference (95% CI)	*P* value
	Domagrozumab	Placebo	Domagrozumab	Placebo		
Muscle volume^a^						
17	71	39	3.92 (2.22, 5.63)	0.98 (− 1.10, 3.06)	2.95 (0.76, 5.13)	0.009
33	70	37	4.30 (2.25, 6.34)	1.38 (− 1.26, 4.02)	2.92 (− 0.05, 5.88)	0.054
49	74	35	3.29 (0.89, 5.68)	− 0.80 (− 3.98, 2.38)	4.09 (0.41, 7.77)	0.030
MVI^a^						
17	71	39	− 3.86 (− 5.15, − 2.57)	− 5.43 (− 7.09, − 3.78)	1.58 (− 0.12, 3.27)	0.068
33	70	37	− 6.80 (− 8.44, − 5.16)	− 9.41 (− 11.61, − 7.21)	2.61 (0.15, 5.07)	0.038
49	74	35	− 10.15 (− 12.09, − 8.20)	− 13.36 (− 16.02, − 10.70)	3.21 (0.13, 6.28)	0.041
Inter/intramuscular fat volume^a^						
17	71	39	18.17 (13.42, 22.93)	19.62 (13.52, 25.72)	− 1.45 (− 7.89, 5.00)	0.66
33	70	37	30.44 (24.42, 36.46)	35.57 (27.51, 43.64)	− 5.13 (− 14.28, 4.02)	0.27
49	74	35	45.14 (37.22, 53.05)	48.14 (37.25, 59.04)	− 3.01 (− 15.86, 9.85)	0.64
T2 muscle bundle, ms^b^						
17	71	39	1.31 (0.66, 1.96)	2.12 (1.30, 2.94)	− 0.81 (− 1.65, 0.03)	0.060
33	66	37	2.57 (1.83, 3.32)	3.69 (2.73, 4.64)	− 1.11 (− 2.16, − 0.07)	0.037
49	70	34	3.01 (2.08, 3.93)	5.51 (4.27, 6.75)	− 2.51 (− 3.93, − 1.08)	0.001
T2 lean muscle, ms^b^						
17	71	39	0.99 (0.33, 1.65)	1.39 (0.56, 2.21)	− 0.40 (− 1.26, 0.46)	0.36
33	66	37	1.70 (0.98, 2.42)	2.99 (2.07, 3.92)	− 1.29 (− 2.30, − 0.29)	0.012
49	70	34	1.89 (1.01, 2.77)	3.84 (2.67, 5.02)	− 1.96 (− 3.30, − 0.61)	0.005
Percent non-elevated voxels, %^b^						
17	71	39	− 2.11 (− 3.09, − 1.12)	− 3.47 (− 4.71, − 2.23)	1.36 (0.08, 2.64)	0.038
33	66	37	− 3.67 (− 4.88, − 2.46)	− 6.06 (− 7.62, − 4.50)	2.39 (0.64, 4.14)	0.008
49	70	34	− 4.65 (− 6.07, − 3.23)	− 8.00 (− 9.93, − 6.07)	3.35 (1.13, 5.58)	0.004
Fat fraction muscle bundle, %^b^						
17	61	32	2.54 (1.73, 3.34)	3.91 (2.81, 5.02)	− 1.38 (− 2.50, − 0.25)	0.017
33	56	29	4.54 (3.35, 5.73)	6.33 (4.66, 7.99)	− 1.78 (− 3.70, 0.13)	0.068
49	61	29	6.42 (4.95, 7.88)	8.55 (6.47, 10.64)	− 2.14 (− 4.59, 0.31)	0.087
Fat fraction lean muscle, %^b^						
17	61	32	0.79 (0.33, 1.25)	0.90 (0.28, 1.51)	− 0.11 (− 0.72, 0.51)	0.73
33	56	29	1.08 (0.55, 1.62)	1.55 (0.82, 2.28)	− 0.47 (− 1.25, 0.32)	0.24
49	61	29	1.60 (0.93, 2.27)	2.25 (1.31, 3.19)	− 0.65 (− 1.72, 0.42)	0.23

*%CFB* percent change from baseline, *CFB* change from baseline, *CI* confidence interval, *MVI* muscle volume index

^a^%CFB

^b^CFB

#### T2 mapping measures

There were significant differences between the domagrozumab and placebo groups at weeks 33 and 49 in mean T2 muscle bundle (week 33, difference − 1.11 ms; 95% CI − 2.16, − 0.07; *P* = 0.037; week 49, difference − 2.51 ms; 95% CI − 3.93, − 1.08; *P* = 0.001; Table 2 and Fig. 2d), and mean T2 lean muscle (week 33, difference − 1.29 ms; 95% CI − 2.30, − 0.29; *P* = 0.012; week 49, difference − 1.96 ms; 95% CI − 3.30, − 0.61; *P* = 0.005; Table 2 and Fig. 2e). Significant differences between the domagrozumab and placebo groups in percent non-elevated voxels were observed at week 17 (difference 1.36%; 95% CI 0.08, 2.64; *P* = 0.038), 33 (difference 2.39%; 95% CI 0.64, 4.14; *P* = 0.008), and week 49 (difference 3.35%; 95% CI 1.13, 5.58; *P* = 0.004; Table 2 and Fig. 2f).

#### Thigh fat fraction

At week 17, there was a significant difference between the domagrozumab and placebo groups in the mean fat fraction of the muscle bundle (difference − 1.38%; 95% CI − 2.50, − 0.25; *P* = 0.017); however, the effect was not significant at week 33 (difference − 1.78%; 95% CI − 3.70, 0.13; *P* = 0.068) or week 49 (difference − 2.14%; 95% CI − 4.59, 0.31; *P* = 0.087; Table 2 and Fig. 2g). There was no significant difference between the domagrozumab and placebo groups in the mean fat fraction of lean muscle at any time point (Table 2 and Fig. 2h).

#### Correlative analysis of key MRI endpoints with change in 4SC time and NSAA score

All key MRI endpoints at week 49 were significantly correlated with 4SC time at week 97 (Table 3). The %CFB in muscle volume and MVI, and change in percent non-elevated voxels, at week 49 were negatively correlated with CFB in 4SC time at week 97. By comparison, %CFB in inter/intramuscular fat volume, and CFB in mean T2 muscle bundle, mean T2 lean muscle, mean fat fraction of the muscle bundle, and mean fat fraction of lean muscle were positively correlated with CFB in 4SC time at week 97. Participants who had an increase in muscle volume > 2.45% from baseline over the first 49 weeks performed better on 4SC after 97 weeks (8.88 %CFB) compared with participants who had marginal muscle volume gains or a decrease in muscle volume over 49 weeks (< 2.45 %CFB) leading to an increase in 4SC after 97 weeks (94.10 %CFB). Regression tree analyses on all other MRI endpoints detected a difference between the two subgroups in percent change in 4SC of a similar magnitude as was seen with muscle volume, suggesting that quantitative MRI measures after 49 weeks may predict 4SC changes after 97 weeks.

**Table 3:**
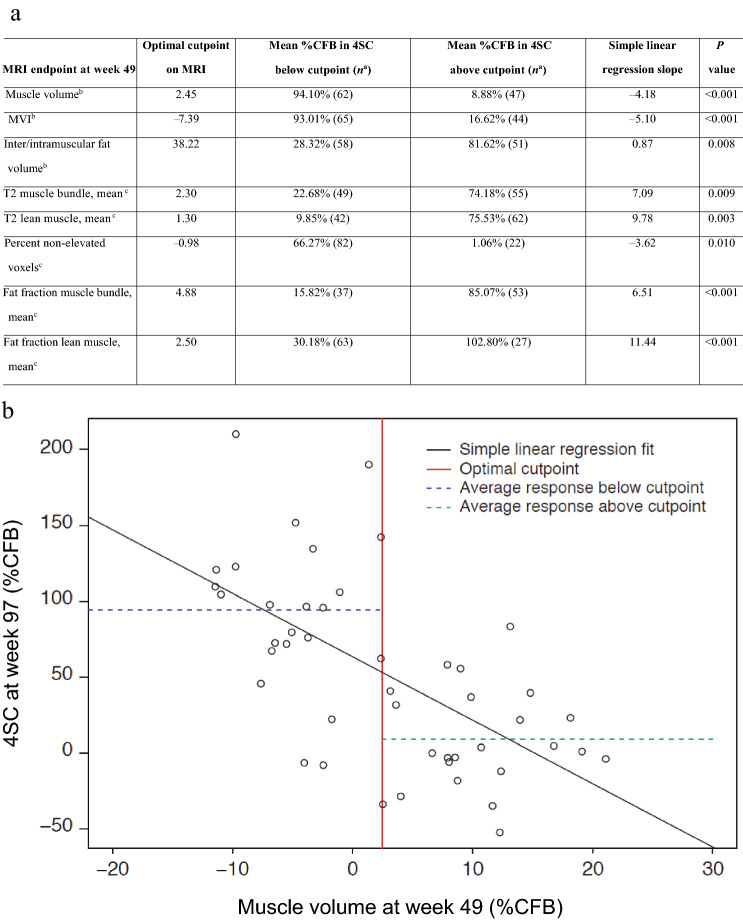
**a** Summary of linear regression and regression tree analyses of MRI endpoints at week 49 vs. 4SC at week 97, and **b** example regression scatter plot showing the relationship between muscle volume at week 49 and 4SC at week 97

Unscheduled and early termination readings have been excluded from the presentation. Missing values have been excluded before calculating estimates by linear regression model. Optimal cutpoints were determined using regression trees allowing for a single cutpoint on the MRI parameter

*4SC* four-stair climb, *%CFB* percent change from baseline, *BL* baseline, *CFB* change from baseline, *MRI* magnetic resonance imaging, *MVI* muscle volume index

^a^Number in subgroup

^b^%CFB

^c^CFB

All key MRI endpoints at week 49 were also significantly correlated with NSAA at week 97 (Table 4). The %CFB in muscle volume and MVI, and change in percent non-elevated voxels, at week 49, were positively correlated with CFB in NSAA at week 97. By comparison, %CFB in inter/intramuscular fat volume, and CFB in mean T2 lean muscle, mean T2 muscle bundle, mean fat fraction of the muscle bundle, and mean fat fraction of lean muscle were negatively correlated with CFB in NSAA at week 97. Participants who had a large percent change in muscle volume at week 49 (>7.92 %CFB) performed better on NSAA at week 97 (− 1.37 CFB) compared with participants who had a small percent change in muscle volume (leading to − 8.17 CFB on NSAA). Regression tree analyses on all MRI endpoints yielded a difference of at least 5 points on NSAA CFB to week 97 between the two subgroups split by the associated MRI endpoint.

**Table 4:**
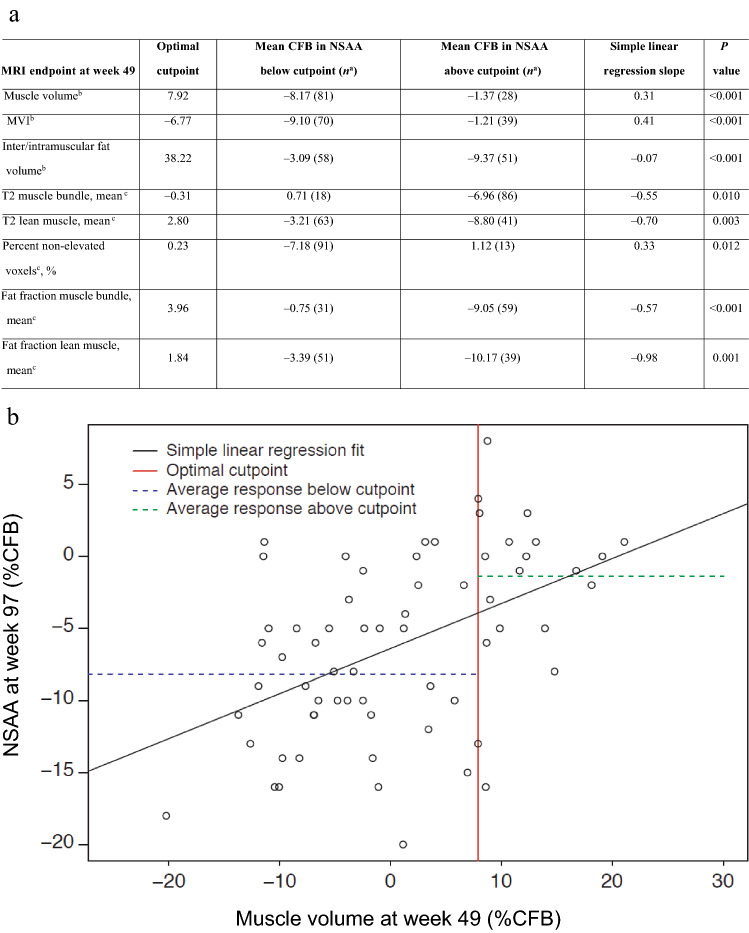
**a** Summary of linear regression and regression tree analyses of MRI endpoints at week 49 vs. the NSAA at week 97, and **b** example regression scatter plot showing the relationship between muscle volume at week 49 and NSAA at week 97

^a^Number in cohort

^b^%CFB

^c^CFB

Unscheduled and early termination readings have been excluded from the presentation. Missing values have been excluded before calculating estimates by linear regression model. Optimal cutpoints were determined using regression trees allowing for a single cutpoint on the MRI parameter

*%CFB* percent change from baseline, *BL* baseline, *CFB* change from baseline, *MRI* magnetic resonance imaging, *MVI* muscle volume index, *NSAA* North Star Ambulatory Assessment

#### Time to loss of ambulation

**Fig. 3:**
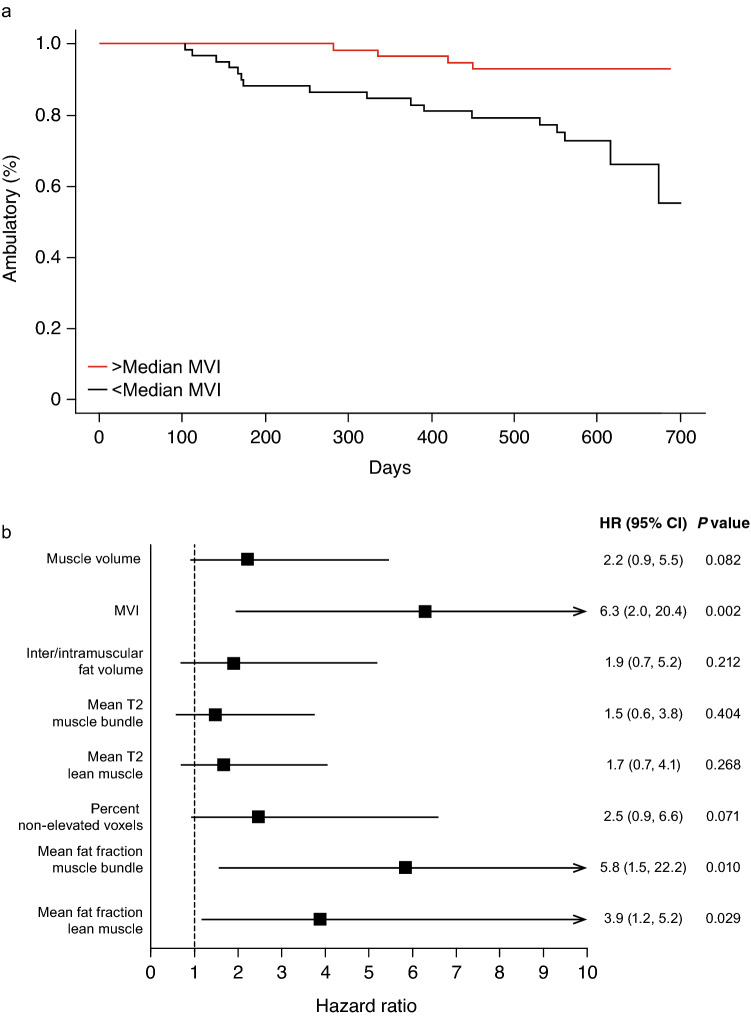
MRI biomarkers at baseline and relation to loss of ambulation. **a** Kaplan–Meier plot showing percent of participants who were ambulatory over the 2-year study. Participants were stratified based on their baseline MVI with the red curve showing participants above the median MVI, and the black curve showing participants below the median MVI. The hazard ratio for loss of ambulation based on MVI at baseline was 6.3. There was a significant difference between the two groups (*n* = 60 per group, *P* = 0.0002); **b** forest plot showing the individual hazard ratios for loss of ambulation based on all MRI-based biomarkers at baseline. In each case, less favorable MRI values at baseline were associated with a higher probability of loss of ambulation. *MRI* magnetic resonance imaging, *MVI* muscle volume index

Twenty-two participants lost ambulation during the study. All baseline MRI parameters, when stratified by their median value, showed that a less favorable MRI value at baseline was associated with a higher probability of loss of ambulation (Fig. 3). Hazard ratios ranged from 1.5 to 6.3, and MVI, mean fat fraction of muscle bundle, and mean fat fraction of lean muscle were statistically significant (all *P* < 0.05). Time-varying covariate analyses yielded a statistically significant relationship between MRI over time and time to loss of ambulation, except for mean T2 muscle bundle and inter/intramuscular fat volume.

#### Bivariate analysis of the relationship between the CFB in MRI parameters at week 49 and the CFB in NSAA at week 97

**Fig. 4:**
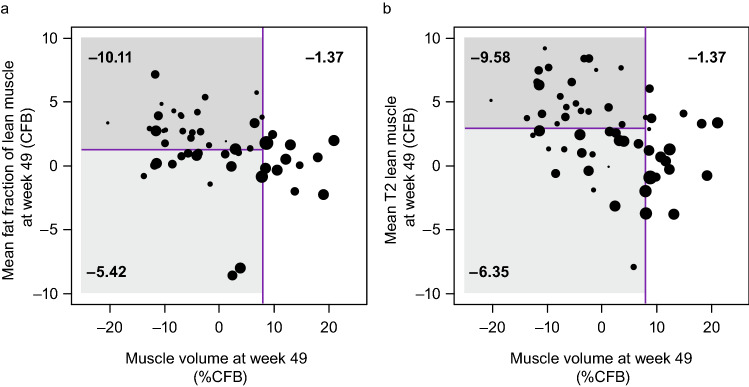
Bivariate analysis of the relationship between the CFB in NSAA and the CFB to week 49 in combined MRI parameters. **a** Muscle volume and mean fat fraction of lean muscle; **b** muscle volume and mean T2 lean muscle. Each point in the figures represents a single subject. The size of the point is proportional to the CFB in NSAA score at week 97 for that subject, with larger points representing a smaller decline in NSAA score. The numbers in the red boxes are the mean NSAA CFB within each region of the plot. *%CFB* percent change from baseline, *CFB* change from baseline, *MRI* magnetic resonance imaging, *NSAA* The North Star Ambulatory Assessment

Participants with a greater percent increase in muscle volume at week 49 (> 7.92%) reported a smaller reduction in NSAA after 97 weeks (− 1.37 CFB) irrespective of change in mean fat fraction of lean muscle at week 49 or change in mean T2 lean muscle (Fig. 4). Participants who had smaller increases in muscle volume (< 7.92%), or those who lost muscle volume over 49 weeks had better functional outcomes if they had preserved muscle quality as indicated by lower change in fat fraction (− 5.42 CFB in NSAA compared with − 10.11) or lower change in mean T2 values (− 6.35 CFB in NSAA compared with − 9.58).

#### MRI parameters vs. age

To demonstrate the expected change over different age ranges, all MRI parameters were plotted vs. participant age (Supplementary Fig. 1). These plots do not distinguish treatment effects and only showed the change in the participant cohort over the 48-week treatment period. All imaging measures, except for muscle volume, show a declining trend with participant’s advancing age.

## Discussion

Following 48 weeks of treatment, MRI measures detected significant changes in muscle volume, MVI, and T2 mapping measures in boys treated with domagrozumab vs. placebo. Muscle volume measures demonstrated an anabolic effect of treatment with domagrozumab, consistent with the expected mechanism of action of a myostatin inhibitor and with preclinical studies in the *mdx* mouse. Muscle volume index, reflecting the percent of total thigh tissue that was lean muscle, also demonstrated a treatment effect with a higher MVI measure in boys treated with domagrozumab for 48 weeks.

T2 mapping measures that evaluated mean T2 relaxation times in lean muscle and the thigh muscle bundle indicated that treatment with domagrozumab reduced the average T2 relaxation time. Lower T2 relaxation times, reflecting decreased fat infiltration, edema, and/or inflammation, suggest that domagrozumab may have helped reduce muscle damage. This finding is further supported by the higher percent non-elevated voxels values observed in boys treated with domagrozumab. As shown previously, the distribution of T2 mapping values spreads with disease progression [16]. The reduction in elevated voxels supports the pharmacodynamic effect observed with domagrozumab treatment.

Overall, domagrozumab appeared to slow muscle degeneration and fatty infiltration as evaluated using T2 mapping and fat fraction analysis, while increasing volume of lean muscle tissue. Although differences in mean CFB of fat fraction measures were not statistically significant for domagrozumab vs. placebo at week 49, there were directionally favorable changes. Differences between treatment groups were detected over the 49-week treatment period; however, dose-dependent differences were not noted following the dose escalation at weeks 17 and 33. Despite muscle MRI measures demonstrating that domagrozumab had an effect on delaying disease progression, the phase 2 trial did not report any statistically significant differences between domagrozumab and placebo in functional changes as evaluated by 4SC (primary efficacy endpoint) or NSAA (secondary efficacy endpoint) at week 49 [32, 33]. As a result of the lack of clear functional benefit following treatment with domagrozumab, the trial was subsequently discontinued early.

It has been suggested that subtle changes in muscle may precede functional changes in boys with DMD. This is supported by recent studies demonstrating that MRI measures may be a useful tool to inform longer term functional changes [2, 24]. Linear regression analyses were performed to investigate the relationship of MRI changes at week 49 vs. 4SC and NSAA changes at week 97. These analyses demonstrated that thigh muscle volume, MVI, inter/intramuscular fat volume, T2 mapping measures, and thigh fat fraction measures were significantly correlated with 4SC and NSAA measures after 97 weeks. This finding supports the concept that MRI changes may be observed in advance of functional changes.

In addition to assessing the correlations between week 49 MRI changes and week 97 functional changes, optimal cutpoints, which separated each biomarker into two subgroups, were identified using regression tree methods. The optimal cutpoint provides a threshold for the given imaging biomarker, which maximizes the difference between functional outcomes after 97 weeks. These optimal cutpoints yielded an average difference of 67% between subgroups on 4SC and at least a 5-point difference on NSAA. The regression tree analysis further supports the use of quantitative MRI measures as biomarkers for detecting early treatment effects in DMD.

Despite the small number of participants who experienced loss of ambulation during the study, hazard ratios indicated that participants with a more favorable baseline MRI disposition are less likely to experience loss of ambulation over a 2-year period. The analyses of time to loss of ambulation using MRI parameters as time-varying covariates suggests that unfavorable changes in MRI parameters over time are associated with an increased risk of losing ambulation.

Using bivariate analysis, we conducted a proof-of-concept analysis to investigate if the relationship between imaging and functional assessments can be strengthened by combining multiple imaging biomarkers, with the CFB in NSAA after 97 weeks used as the measure of function. Muscle volume was combined with either fat fraction of lean muscle or mean T2 of lean muscle to examine sensitivity by combining measures of lean muscle volume and lean muscle quality (as evaluated by T2 or fat fraction measures). The result of this analysis suggests that increases in muscle volume > 7.92% after 49 weeks leads to better preservation of NSAA scores after 97 weeks. For boys who had muscle volume increases of < 7.92%, muscle quality had to be preserved to maintain function. In other words, boys with low muscle volume and poor muscle quality tended to have the most significant declines in physical function as assessed by NSAA after 97 weeks.

To apply MRI biomarkers in clinical trials, it will be important to understand the anticipated change over time within a group of subjects. To this end, we plotted participant age vs. all imaging biomarkers. Although treatment effects were not considered in these plots, the expected change in different biomarkers across age groups can be inferred. Muscle volume appeared to be relatively independent of participants’ age. This finding was initially suggested by looking at baseline correlations between participants’ age and muscle volume [29], and is further supported by looking at longitudinal measures. This finding reflects two concomitant processes in boys with DMD, namely muscle growth with muscle degeneration and fatty infiltration as the disease progresses. Despite increasing thigh length over 49 weeks, the increasing fatty infiltration and muscle wasting leads to a relatively constant lean muscle volume across the age range studied. MVI and fat fraction measures demonstrated a more “sigmoidal” shape, indicating that MRI measures in boys aged 8–10 years may progress faster than in younger or older boys. This is consistent with previous natural history studies evaluating boys with DMD over multiple years [2, 24].

This study demonstrates that quantitative MRI measures can be objective biomarkers that can be included in large, multicenter, international clinical trials. Standardization across MRI equipment at clinical trial sites is feasible and image analysis can be scaled for use in clinical trials. Furthermore, the study demonstrates the successful use of MRI analyses in pediatric participants (6 to < 16 years of age) without the need for sedation, removing a problematic feature when planning MRI assessments as part of pediatric clinical trials [19]. Despite the multicenter design, the young population (mean age < 9 years), and the potential for cognitive impairment and behavioral comorbidities affecting participant cooperation, over 97% of the MRI scans received were evaluable. The most common reason for a scan being non-evaluable was participant motion.

The original clinical trial was not specifically designed to demonstrate imaging endpoints as predictive biomarkers, nor was it a prespecified goal of the study design, limiting its generalizability. To truly establish MRI measures as predictors of functional changes in subjects with DMD, additional studies would need to be performed with this explicit goal in mind. The crossover design was particularly limiting in fully establishing quantitative MRI measures as predictive biomarkers, as there could have been slight alterations to functional performance after participants altered their therapeutic course. An additional limitation is that not all participants were followed until their week 97 visit. Although the study was terminated early for lack of efficacy, a few participants may not have had long-term functional assessments due to loss of ambulation or early discontinuation, both of which may introduce some bias into the assessment of week 97 outcomes.

Overall, this study demonstrates that MRI-based biomarkers can detect small changes in muscle volume and quality and can be incorporated into multicenter trials. The exact threshold of change needed to induce a functional benefit is still under investigation; however, these preliminary analyses suggest a relationship between changes in MRI-based biomarkers after 49 weeks and functional changes (NSAA and 4SC) after 97 weeks. These results also suggest that MRI-based biomarkers at baseline can be used to identify participants at higher risk of loss of ambulation over a clinical trial monitoring period.

### Conclusions

In DMD, quantitative MRI measures can be viable biomarkers to help inform clinical trials and have the potential to predict future functional changes. The standardized acquisition methods used were scalable in a multicenter international study and may guide future clinical trials to enable the detection of subtle changes in muscle. Despite the imaging results reported in this analysis, at the time of primary study completion, the totality of evidence did not support clear clinical benefit with domagrozumab in DMD.

## Supplementary Information

Below is the link to the electronic supplementary material.

Supplementary file1 (EPS 2625 KB)

## Data Availability

Upon request, and subject to review, Pfizer will provide the data that support the findings of this study. Subject to certain criteria, conditions and exceptions, Pfizer may also provide access to the related individual de-identified participant data. See https://www.pfizer.com/science/clinical-trials/trial-data-and-results for more information.
